# Accounting For Alignment Uncertainty in Phylogenomics

**DOI:** 10.1371/journal.pone.0030288

**Published:** 2012-01-17

**Authors:** Martin Wu, Sourav Chatterji, Jonathan A. Eisen

**Affiliations:** 1 Department of Biology, University of Virginia, Charlottesville, Virginia, United States of America; 2 Genome Center, University of California, Davis, California, United States of America; 3 Section of Evolution and Ecology, College of Biological Sciences, University of California, Davis, California, United States of America; 4 Department of Medical Microbiology and Immunology, School of Medicine, University of California, Davis, California, United States of America; University of Florida, United States of America

## Abstract

Uncertainty in multiple sequence alignments has a large impact on phylogenetic analyses. Little has been done to evaluate the quality of individual positions in protein sequence alignments, which directly impact the accuracy of phylogenetic trees. Here we describe ZORRO, a probabilistic masking program that accounts for alignment uncertainty by assigning confidence scores to each alignment position. Using the BALIBASE database and in simulation studies, we demonstrate that masking by ZORRO significantly reduces the alignment uncertainty and improves the tree accuracy.

## Introduction

Multiple sequence alignment is critical for many biological studies making use of sequence data. For evolutionary analysis, columns in multiple sequence alignments are hypothesized to contain homologous residues in different sequences. This is known generally as “positional homology”. The assignment of positional homology can be problematic, however. It follows that the quality of a sequence alignment has a large impact on the final phylogenetic trees [1], [2], [3], [4], [5], [6], so much so the inferred phylogeny may be more dependent upon the methods of alignment than on the methods of phylogenetic reconstruction [1], [5], [6], [7], [8], [9]. This is especially true for highly divergent sequences whose alignments are more difficult and less consistent.

A plethora of programs developed recently have led to significant improvement of the overall alignment accuracy [10], [11], [12], [13], [14], [15], [16], [17], [18]. Despite this, the alignment uncertainty in typical real sequence dataset continues to cause problems for phylogenetic studies. In one striking example, Landan and Graur showed that aligning protein sequences from the N-terminus (the head) to the C-terminus (the tail) can, and in many cases do, produce alignments that were highly different from the same sequences aligned from the C- to the N-terminus, despite that identical sequences and alignment algorithms were used [9]. This is thought to be caused by the presence of multiple equally optimal but distinct solutions during the alignment process. To deal with the equivocality, alignment programs either intentionally or not, end up making arbitrary decisions that can lead to significantly different alignments [19] and incongruent phylogenies [9]. Alignment uncertainty has become even a bigger problem in the era of phylogenomics, when phylogenetic analyses of thousands of genes are routinely carried out automatically without accounting for the alignment reliability. For example, using genomic data from seven yeast species, Wong and colleagues demonstrated that variations in sequence alignments produced by different alignment methods were significant enough to lead to different phylogenetic conclusions – 46.2% of the 1,507 genes had one or more differing trees depending on the alignment method used [20]. In one particularly striking case, seven alignments of a gene family produced six different phylogenies of seven yeast species [20].

Several metrics have been introduced to help assess the overall alignment quality [21], [22], [23], [24]. Although appearing in slightly different forms, they all basically quantitatively measure the differences between alignment variants of the same set of sequences and use these scores to evaluate the overall alignment quality. The underlying assumption in this approach is that if the alignment fluctuates considerably with different methods (and thus can be considered unstable), this implies that the alignment is difficult and might be of poor quality. When compared to high quality reference alignments, the overall sensitivity (defined as the number of correctly aligned residues divided by the number of residues aligned in the reference alignment) and specificity (defined as the number of correctly aligned residues divided by the number of residues aligned by a particular alignment program) of an alignment can be calculated as well. For example, using simulated datasets and the SABmark database [25], Pachter and his colleagues benchmarked several commonly used alignment programs. They found that all were heavily biased toward maximizing the sensitivity at the expense of the specificity [26], i.e., although many residues were correctly aligned, it is also the case that a large fraction of the characters were aligned incorrectly. In other words, the assumption of positional homology was invalid for many of the aligned positions.

A critical, yet largely unsolved problem in the field is how to assess the quality of the alignment at each individual position, (i.e., the validity of the assignment of positional homology). Knowing the quality of the individual position is important as “poorly” aligned columns are more likely to contribute noise than signal. Detecting and removing ambiguously aligned regions, a step known as masking and trimming in molecular phylogenetics, increases the signal-to-noise ratio and improves the discriminatory power of phylogenetic methods [27], [28], [29]. Traditionally, masking and trimming of regions thought to be poorly aligned were done as part of manual curation process. Such manual efforts are not only subjective but also impractical in large-scale phylogenetic inferences. Positions with gaps are often considered unreliable and therefore are trimmed. However, it has been shown that trimming by simply removing positions that contain gaps results in excessive loss of informative sites and does not necessarily lead to better trees [20], [30].

GBLOCKS is currently the most frequently used masking program that attempts to assess the quality of alignment position by position. It calculates the degree of conservation for each aligned position and then uses it to select conserved “blocks” for further analyses [27]. However, positions with low conservation scores could still be homologous (e.g., fast evolving sites). Such sites might contain useful phylogenetic information, sometimes more so than these highly conserved positions. To overcome this limitation, GBLOCKS tries to “rescue” these poorly conserved but potentially homologous positions as long as they belong to a block flanked by highly conserved columns at both ends and satisfy a set of ad hoc rules (e.g., the maximum number of contiguous nonconserved positions allowed is 8 and the minimum length of a block is 10). However, in real alignments, homologous regions are not always punctuated by highly conserved columns. In addition, these ad hoc rules are quite arbitrary with little theoretic support.

Several alternative masking programs were recently developed. For example, ALISCORE identifies low quality regions within protein alignments based on Monte Carlo resampling within a sliding window [31]. A position is considered to be noisy if its similarity score is not significantly better than random. SOAP [32] and GUIDANCE [33] use a different approach. They identify ‘unstable-hence-unreliable’ alignment positions by comparing a set of suboptimal alignments against a user-defined reference alignment. Although more objective than GBLOCKS, there are limitations with these recently developed programs. For example, ALISCORE can only assess the positional homology within the context of a local sliding window but not in the entire alignment landscape. For SOAP and GUIDANCE, it is not clear whether the limited set of alignment variants are general enough to take into account all alignment errors. Another line of effort to take alternative alignments into account is to estimate the alignment and phylogenetic tree simultaneously [34]. However, this approach is computationally intensive and is not practical for large-scale phylogenetic tree reconstructions.

Here we introduce ZORRO, a probabilistic masking program that objectively measures the quality of the alignment at each individual position. ZORRO uses a pair Hidden Markov Model (pair-HMM) to model the sequence evolution and uses the model to calculate the posterior probabilities that residues of a column are correctly aligned or homologous. In this paper, we first introduce the theoretical motivation behind ZORRO. Then using simulated sequences, we demonstrate that ZORRO outperforms other masking programs in terms of the masking sensitivity and specificity. We further show that masking by ZORRO reduces the alignment uncertainty that in turn leads to more accurate phylogenetic trees for relatively long and divergent sequences.

## Results

### Algorithm overview

Our goal was to develop an objective metric that measures the confidence of the positional homology, the core assumption that underlies most molecular phylogenetic inferences. A sequence alignment is not an observation but rather a hypothesis wherein a particular alignment is selected as the best from multiple options. Consequently, when evaluating the quality of two aligned residues, it would be best to do it in the context of all possible alignments and not just one single alignment usually obtained. The idea is that if two residues are truly homologous, we assume that they will also align in most of the alternative alignments. On the other hand, if the match of the two residues is not reliable, then the likelihood that they appear together in the alignment space should be low. This type of strategy has been used empirically to extract good quality sub-alignments from a limited set of alignment variants and combine them into a new, often improved alignment [35], [36], and to assess the positional alignment quality [33].

Pair hidden Markov model (HMM) provides an ideal probabilistic framework for modeling alignment problems [13], [37], [38]. Using pair HMM, the posterior probability of two residues being aligned in all possible alignments can be readily calculated using the forward-backward algorithm of the hidden Markov model. ZORRO implements a variant of standard pair HMM. It calculates the probability of all alignments that pass through a specified matched pair of residues. It then compares this value with the full probability of all alignments of the pair of sequences. If the ratio (posterior probability) is close to 1, then the match is highly reliable. If the ratio is close to 0, then the match is considered to be ambiguous. To assess the confidence of an entire column, ZORRO uses a weighted sum of pairs scheme to sum up the posterior probability of all pairs in the column and assign a confidence score between 0 and 1 to each column. In comparison, profile HMMs calculate the posterior probability for a given input residue aligning with a given alignment column (profile). It has been used to evaluate how well an input residue aligns with the columns that are used to build the profile HMM (e.g., as in HMMER3 package). However, it does not estimate the quality of the aligned columns per se.

The details of the ZORRO algorithm and its implementation are described in the methods section. ZORRO is an open source program written in C and is freely available for download at http://probmask.sourceforge.net.

### Protein sequence simulation

To evaluate the performance of ZORRO and other masking programs, we simulate protein sequence evolution using the program ROSE [39]. We chose to use simulated sequences because we would have the advantage over the real sequences of knowing both the true alignments and the true phylogenies. We therefore not only can evaluate the performance of masking programs on the sequence alignment directly, but also can determine whether masking improves the accuracy of phylogenetic reconstructions.

Our simulations were based on a representative set of 31 phylogenetic marker genes that are broadly conserved among bacterial species [40]. They are single-copy housekeeping genes with variable sequence lengths and evolutionary rates, and therefore represent good test cases for our simulation study. To best replicate the biological characteristics of these proteins, we simulated based on a bacterial ‘genome tree’ that was inferred from the concatenation of these 31 genes [41], and used site-specific substitution probabilities empirically inferred for each gene to mimic their natural sequence motifs. Additionally, the guide trees were scaled so that the evolutionary rates of the simulated sequences matched those of the original marker genes. For each marker gene, 100 simulations were run.

### The sensitivity and specificity of ZORRO masking

We used the simulated sequences to benchmark the performance of masking. After removing gaps from the true alignment, we realigned the sequences using MAFFT [16] and measured the confidence of each aligned column using ZORRO, GBLOCKS, ALISCORE or GUIDANCE. By comparing the MAFFT alignments with the true reference alignments, we evaluated the performance of masking in terms of the sensitivity and specificity. We define the sensitivity as the fraction of correctly aligned residue pairs that have been marked as reliable by the masking programs. We define the specificity as the fraction of incorrectly aligned residue pairs that have been marked as of low quality. A good masking program should have both high sensitivity and specificity in order to maximize the signal-to-noise ratio of the masked sequence data.

**Figure 1:**
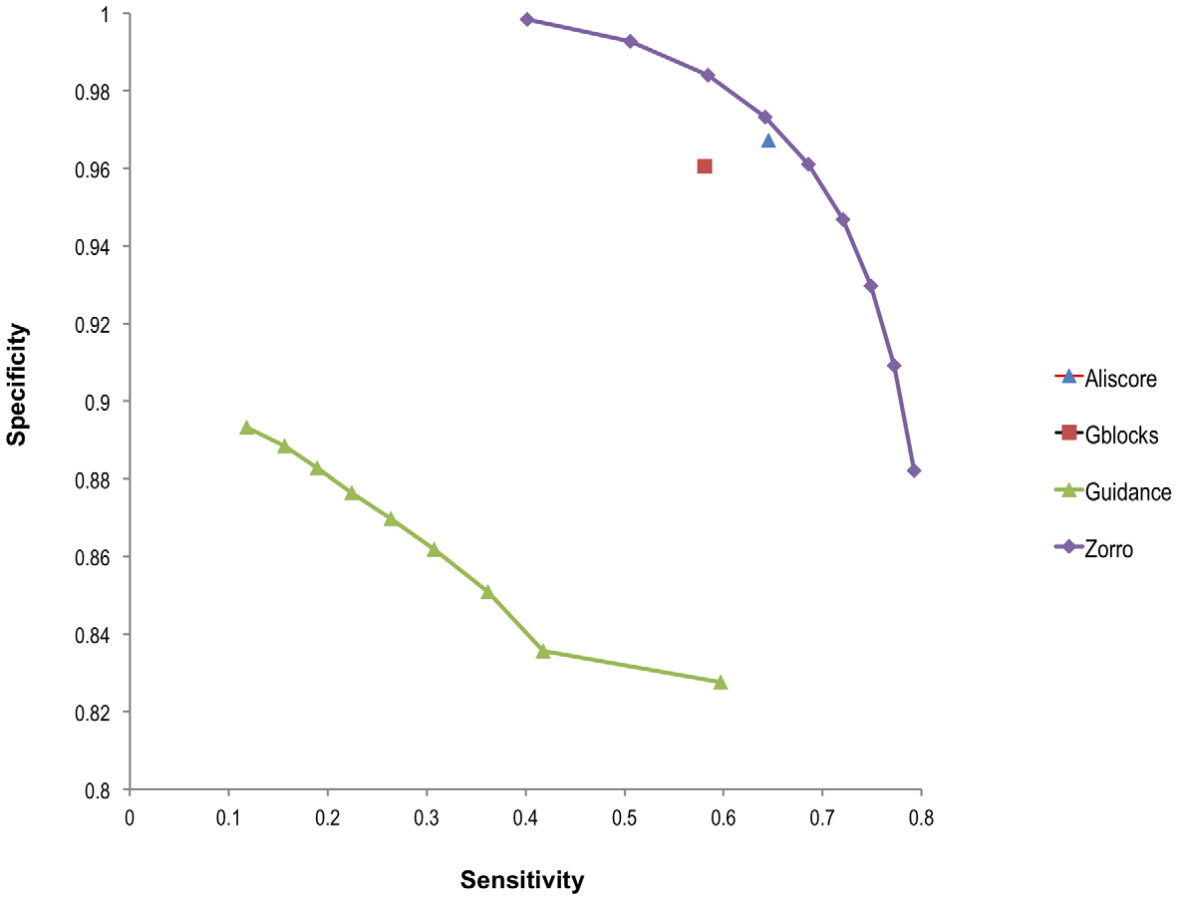
The sensitivity and specificity of alignment masking programs. Each data point in the ZORRO and GUIDANCE series represents one probability cutoff score that was used to mask the alignment. From left to right: 0.9 to 0.1 in the interval of 0.1.

Figure 1 shows the sensitivity and specificity of ZORRO masking using different probability cutoff values. Columns with probability scores equal to or above a certain cutoff were marked as reliable. Otherwise, they were considered as of low quality. As expected, the sensitivity and specificity are negatively correlated – increasing the stringency of the cutoff increases the specificity but decreases the sensitivity of masking, and vice versa. A cutoff of 0.4, 0.5 or 0.6 seemed to all offer a good balance of specificity and sensitivity. A probability cutoff value of 0.4 was therefore used in the subsequent analyses.

Figure 1 shows that ZORRO outperformed the other masking programs. It shows that for the same level of sensitivity, ZORRO provided better specificity than GBLOCKS, ALISCORE and GUIDANCE. GUIDANCE performed poorly in comparison to the other programs, regardless of the cutoff scores used to mask the alignments. Similar results were obtained from alignments produced using other methods such as CLUSTALW (data not shown).

### ZORRO reduces the alignment uncertainty

Given that ZORRO performs well relative to the other masking programs, our next question was to determine if it produced output that could then be used to clean up alignments with reduced uncertainty. This would be beneficial because as discussed above, uncertainty in alignments can lead to phylogenetic inference errors. Ambiguous columns typically do not persist between different alignment treatments and will cause alignments to differ. By measuring the extent of differences between alignments, the uncertainty in alignments can be quantified. The head-or-tail approach discussed earlier is a simple and elegant way of assessing the alignment uncertainty [9] and is used here.

**Figure 2:**
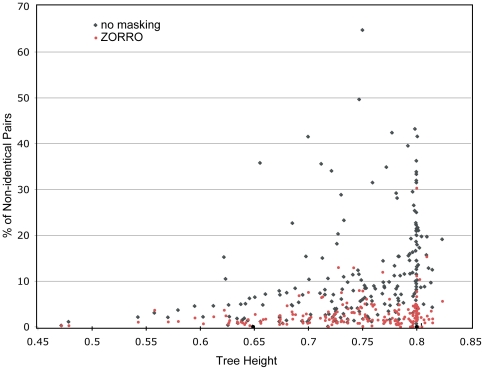
Alignment discrepancies between the head and tail alignments at different sequence divergence levels before (black) and after (red) ZORRO masking. Masking by ZORRO makes the alignments substantially more consistent.

We realigned the 216 protein families in the BALIBASE database from both directions (head or tail) using the program MAFFT. BALIBASE consists of protein families of various size, sequence length and levels of similarity and covers a wide range of structural folds and distinct patterns of evolution histories. For each protein family, we then calculated the proportion of residue pairs that were paired differently (discrepancy fraction) in the head vs. the tail alignments, and used it as a measurement of alignment uncertainty, with or without ZORRO masking. Figure 2 shows the discrepancy fraction as a function of the sequence divergence, which we measured using the average height (from the root to the tips) of the trees that were inferred from these alignments. Not surprisingly, the alignment uncertainty increases with the increasing sequence divergence, as sequences become more difficult to align. Masking by ZORRO, however, produced alignments that were more consistent and substantially reduced the discrepancy fraction from 11.3% to 3.1% on average. A much more pronounced effect of masking was observed on the more divergent sequences. Paired t-test indicated this reduction in alignment uncertainty was highly significant with a P value of 1.99e-32.

### ZORRO improves phylogenetic reconstructions

**Figure 3:**
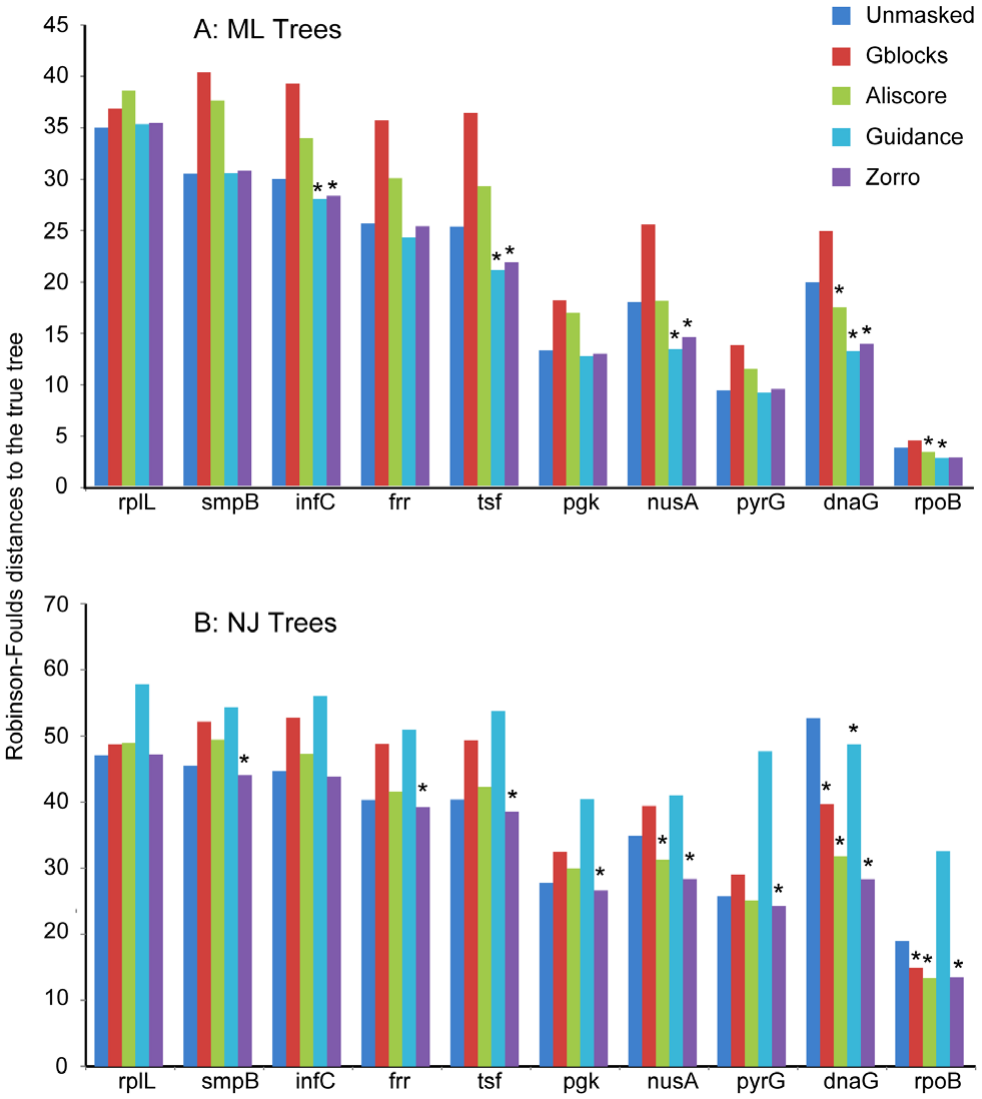
ZORRO improves accuracy of inferred phylogenetic trees. Average Robinson-Foulds distances to the true tree were plotted for each of the ten bacterial phylogenetic markers. The NJ and ML trees were made from simulated sequences that were subjected to five different treatments after being aligned by MAFFT: no masking, masking by GBLOCKS, ALISCORE, GUIDANCE and ZORRO. The asterisk indicates where masking significantly improves the tree accuracy (100 replicates, sign test P<0.05).

Simulation studies provided further support that ZORRO benefit the downstream phylogenetic reconstructions (Figure 3). For the NJ trees, ZORRO significantly improved the accuracy in 8 out of 10 genes. The improvement is less pronounced in the ML trees, with significant improvement observed in half of the genes and no significant changes in the other half of genes. Therefore, it seems that NJ trees benefit more than ML trees from the alignment masking. One possible explanation is that the ML method, by accommodating the evolution rate heterogeneity (i.e., with gamma distribution), could account for the alignment uncertainty to a certain degree.

Under the conditions tested in our study, ZORRO consistently outperformed GBLOCKS, ALISCORE and GUIDANCE in NJ trees. GBLOCKS, ALISCORE and GUIDANCE improved the NJ trees when the alignments were relatively long (e.g., DnaG, RpoB) but had little or even negative impact when the alignments were short. For ML trees, ZORRO outperformed GBLOCKS and ALISCORE and was on a par with GUIDANCE. One advantage of using ZORRO and GUIDANCE is that they assign a confidence score to each individual position, so it is possible to weigh each alignment position by their confidence scores (e.g., using RAxML's column weight option). Low-quality positions will still contribute to phylogenetic reconstruction but will do so at a much lower weight. In comparison, position-specific weighting is not possible with GBLOCKS and ALISCORE. Consequently, low-quality positions are removed prior to the phylogenetic reconstruction, which can lead to the excessive loss of phylogenetic signal.

**Figure 4:**
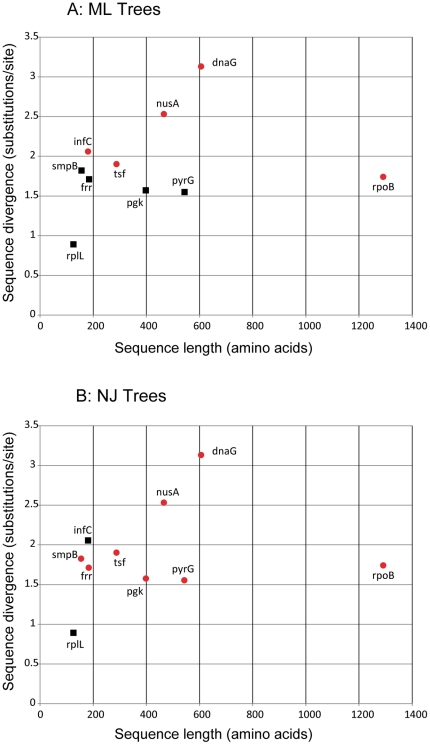
The impact of sequence length and divergence on the performance of ZORRO masking. Red circles indicate that ZORRO significantly improves the tree accuracy while the black squares indicate that no significant improvement was observed. The longer the sequence and the faster the evolutionary rate, the more likely that ZORRO masking will have a beneficial impact on the tree accuracy.

**Figure 5:**
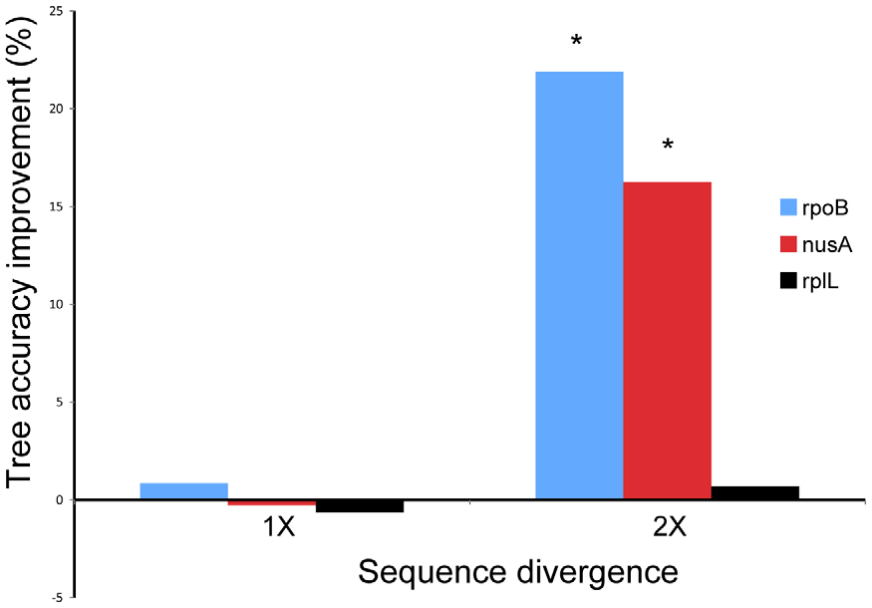
The impact of the sequence divergence on the performance of ZORRO masking. 1× divergence is equivalent to an average distance of 1.1 substitutions/site between a pair of sequences. The Y-axis shows the tree accuracy improvement in terms of the Robinson-Foulds distance: (Distance_unmasked_-Distance_zorro_)/Distance_unmasked_. The asterisk indicates significant improvement (100 replicates, sign test, P<0.05).

Previous studies indicated that the sequence length and substitution rate are two important parameters affecting the outcomes of masking [4], [42]. Figure 4 shows a trend consistent with this observation. The longer the sequence and the faster the evolutionary rate, the more likely that masking will have a beneficial impact. To further test this hypothesis, we selected three genes of different lengths (in amino acids, *rplL*, 126; *nusA*, 466; *rpoB*, 1291) and evolved them at two different substitution rates (Figure 5, 1× and 2×). When sequences are highly conserved, masking does not seem to matter much. This is expected because the fraction of unreliable alignment is small. When sequences are relatively short (e.g., RplL), the benefit of masking is also limited. This is because masking does lead to loss of some phylogenetic signal [4] and this negative impact will be more prominent when the phylogenetic signal is already weak in short sequences. However, for sequences that are reasonably long and diverged (e.g., RpoB and NusA), masking can increase the signal-to-noise ratio and significantly improve the tree accuracy.

## Discussion

Explosive growth of genomic-scale sequence data has presented both opportunities and challenges for large-scale phylogenetic inferences. Somewhat underappreciated by the evolutionary and genomics communities is the impact of alignment uncertainty on the trees. As has been demonstrated in a recent study, not accounting for alignment uncertainty can result in dubious results, only more so for phylogenomic studies [20]. Although many metrics have been introduced to quantify the overall alignment quality, more work need to done in developing objective methods to evaluate the alignment qualities of individual columns.

Using pair HMMs, ZORRO sums up the probability that a particular column would appear over the alignment landscape and thus provides an objective measurement that has an explicit evolutionary model and is mathematically rigorous. Our testing on the BALIBASE database demonstrates that masking by ZORRO reduces the alignment uncertainty. By selecting reliable columns that persist in the alignment space, ZORRO effectively increases the internal consistency of the alignment and thus the ratio of the phylogenetic signal to the random noise. We show that ZORRO outperformed other programs in masking. This was reflected in our tree simulation study where the ZORRO trees constantly outperformed the other trees.

An often-debated issue in the phylogenetics community is whether the removal of ambiguous regions in sequence alignments actually leads to more accurate trees [4], [27], [29], [32], [43]. We demonstrate here that masking by ZORRO significantly improved the tree accuracy as long as the sequences are sufficiently long and divergent. In particular, our simulation study indicates that NJ trees benefit tremendously from masking, as reported previously [44]. Because of its speed, NJ is often preferred over the ML method in large-scale phylogenetic analyses. Therefore, masking by ZORRO is well justified for phylogenomic studies, frequently when thousands of NJ trees are made in a batch. We note that most of the bacterial and archaeal protein families will be more divergent than the phylogenetic markers used in our simulation studies. Thus they should possess sufficient amount of divergence to benefit from masking. It is therefore our recommendation to include the masking step in their phylogenetic analyses.

ZORRO is reasonably fast for small and medium size protein families. For large families, ZORRO provides a sampling option that can be invoked to speed up the process without significantly affecting its performance. It is also possible to use ZORRO to pre-compute a mask for a protein family and reuse it later for phylogenetic analyses of the same protein family using tools such as AMPHORA [40]. In this case, there is no additional computational cost at all for masking.

Columns marked as low quality by ZORRO may still contain useful phylogenetic information. This is because ZORRO confidence score measures the “global” quality of the alignment column, i.e. the quality of the column as a whole. However, “globally misaligned” columns may contain correctly aligned subgroups of sequences that are useful for resolving local phylogenetic relationships. A scoring scheme that incorporates such clade-specific “local accuracy” information should help. We are working on developing these for a future release of ZORRO.

## Materials and Methods

### Implementation of ZORRO

To help describing the ZORRO algorithm, we first introduce some notation. For multiple sequence alignments, N is the number of sequences (S) in the alignment *S_1_, S_2_, … S_N_*. For a column *C* in the alignment, the *N* residues are called *C_1_, C_2_*, *… C_N_*. Finally, if two residues X and Y are aligned, it is denoted by X◊Y.

#### Estimation of pair-wise homology probability

To estimate the probability that two residues in a column are aligned correctly, ZORRO implements a variant of standard pair HMM with the state space as described in Figure 4.2 of [37]. The transition probabilities are adapted from the AVID program [45], whereas the emission probabilities are derived from PAM matrices [46]. In addition to the standard match and gap states, an extra state is introduced to model the long gaps. Given two residues *C_i_* and *C_j_* in a column *C*, we can calculate the posterior probability Pr [Ci◊Cj] that the two residues are aligned under the model:
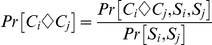
The numerator in the expression is the probability of all alignments in which *C_i_* is aligned to *C_j_*, whereas the denominator is the probability of all alignments between *S_i_* and *S_j_*. For each pair of sequences *S_i_* and *S_j_* in the alignment, these terms can be calculated efficiently for all pairs of residues (and gaps) in quadratic time by using the forward and backward algorithm [37].

#### Scoring Alignment Columns

**Figure 6:**
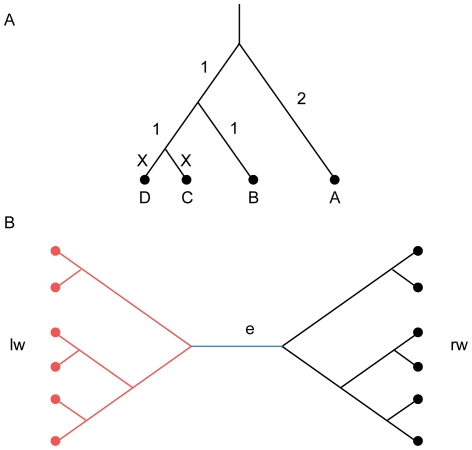
Illustration of the ZORRO weighting scheme. A) The tree contains four nodes A, B, C and D. C and D are very closely related. The paths between pairs A–C and A–D are almost completely overlapping and they each should receive a lower weight to correct for the correlation. Furthermore, closely related pairs such as C–D need to be weighed down as they contain less information about the whole alignment column. B) The branch *e* partitions the sequences (circles) into two subsets (red and black). The weights measuring the correlations among the sequences in each sub-tree are called *lw* and *rw* respectively. Note that these weights are normalized so that they add up to 1.

To score a column in the alignment, the pair-wise posterior probabilities are combined using a weighting scheme described below. The general sum of pairs scheme is not directly suitable for calculating the column score because sequences are not equally related (see Figure 6A for an illustrated example). ZORRO therefore uses a NJ tree to guide a weighted sum of pairs scheme.

An ideal weighted sum of pairs scheme should at least account for the following two factors: 1. Evolutionary distance. The more closely related a pair of sequences are to each other, the less information the corresponding pair of residues carry about the alignment accuracy of the whole column. For example, in Figure 6A, residue pairs from the closely related sequences C and D are always likely to be correctly aligned, but they do not provide much information about the accuracy of the whole alignment column. Therefore, the weight of the pair should account for their evolutionary distance. 2. Correlation between pairs. If two pairs (such as A–C and A–D in Figure 6A) have high overlap, the accuracy of the corresponding residue pairs are highly correlated and need to be accounted for in the weighting scheme as well.

ZORRO uses a simple weighting scheme to account for both factors discussed above. To understand the ZORRO weighting scheme, consider a branch ***e*** in the guide tree (Figure 6B). Each pair *p = S_i_−S_j_* whose path P passes through ***e*** has one sequence each in the left and right subsets. The branch length of ***e***, *w_e_* is divided among all these pairs. Correlation weights **lw** and **rw** are calculated for the sequences in the left and right subsets respectively using a scheme described by Felsenstein [47]. For every pair *p = S_i_−S_j_* whose path P passes through ***e***, let *S_i_* and *S_j_* be in the left and right subset respectively. Then *p* is assigned a share s_pe_ = lw_i_ * rw_j_ of the weight of ***e***.

The weight *w_p_* or *w_ij_* for a pair *p* = *S_i_−S_j_* is set to be the square root of the sum of its share of the weights of all the branches in the path *P* connecting *S_i_* and *S_j_*:
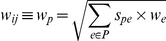
The term *s_pe_* accounts for correlations between pairs that pass though the branch ***e***. Thus the ZORRO weighting scheme accounts for both factors discussed above. These weights are then used to calculate the confidence score *S(C)* for a column *C,* which is the weighted sum of the pair-wise homology probabilities:
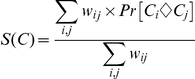



#### Improving Running Time by Sampling Pairs

Since the calculation of the posterior probabilities for each pair of sequence is a quadratic function of the sequence length, the running time of the program is *O(N^2^L^2^),* where *N* is the number of sequences in the alignment and *L* is the length of the longest sequence in the alignment. As protein sequences are comparatively short, the program is thus computationally inexpensive for protein multiple alignments with reasonable number of sequences.

If *N* is large, ZORRO provides the option of reducing the running time without sacrificing much accuracy by calculating posterior probabilities of only a random subset *R* of the *N(N−1)/2* pairs of sequences. The number of pairs in *R* is constrained to be at least *N* and can be specified by the user. The confidence score *S(C)* of each column *C* is calculated by using weighted sums of pairs formula:
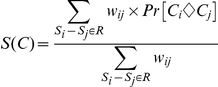
The running time of the modified algorithm is *O(|R|×L^2^)*, where *|R|* is the number of pairs in the set *R* and *L* is the length of the longest sequence in the alignment. The sampling option can be invoked by using “-sample” on the command line in ZORRO.

We run ZORRO on a test dataset with and without the sampling option. 98.2% of columns selected with the sampling option agree with these selected not using the sampling option. Therefore, the sampling option allows us to improve the running time of ZORRO without causing significant changes in the confidence scores.

### Protein Sequence Simulation

We used the program ROSE [39] to simulate protein alignments. ROSE allows for site-specific substitution rates within the sequences, making it possible to model the sequence motifs more realistically than the other sequence simulation programs. We run our simulations based on 31 phylogenetic markers genes that are broadly conserved in bacterial species [40]. Since 22 of the markers are ribosomal proteins of similar size and evolutionary rate, we randomly chose one ribosomal protein gene (*rplL*) as the representative, resulting in using a total of 10 marker genes (*dnaG, frr, infC, nusA, pgk, pyrG, ropB, rplL, smpB* and *tsf*) in our simulation study. The site-specific substitution rate for each gene was estimated using the program rate4site [48] from the marker sequences identified from 720 complete bacterial genomes [41]. To reduce the computational cost, we pruned the ‘genome tree’ of 720 bacterial species [41] (TreeBase, S10956) to 100 taxa (Figure S1) using a greedy algorithm [49] that maximizes the phylogenetic diversity, and then used the pruned tree to guide the sequence evolution. The branch length of the guide tree was scaled for each gene separately so evolutionary rate of the simulated sequences matched that of the natural proteins. The control files that were used by ROSE to simulate the sequences are included in File S1.

### Measure Masking Sensitivity and Specificity

Simulated protein alignments were used to benchmark the performance of the masking programs. After removing the gaps from the simulated alignments, the sequences were realigned using the MAFFT program [16]. For a pair of MAFFT aligned residues, if they were also aligned in the simulated true alignment, we considered the pair to be correctly aligned. Otherwise, we considered the pair to be incorrectly aligned. We then run ZORRO, GBLOCKS, ALISCORE or GUIDANCE on the MAFFT alignments. GBLOCKS was run under the relaxed parameters as described in [4] except that “the minimum length of a block” was relaxed to 3 and “allowed gap positions” was relaxed to “all”. Unless specified, ALISCORE and GUIDANCE were run using their default parameters. The sensitivity of a masking program is then defined as the fraction of correctly aligned pairs that have been marked as reliable by the program. The specificity is defined as the fraction of incorrectly aligned pairs that have been marked as unreliable.

### Simulation Study for Assessing the Tree Accuracy

MAFFT sequence alignments, with or without masking, were used to reconstruct neighbor-joining (NJ) trees using PHYML [50] and maximum likelihood (ML) trees using RAxML [51]. The columns masked as low quality were removed prior to the phylogenetic analysis, except for ZORRO and GUIDANCE masked alignments where the columns were weighted by the confidence scores using RAxML's column weight option. The ML trees were made with the GAMMA+I+JTT model, with the gamma distribution parameters and the proportion of invariable sites estimated by the program itself. 100 replicates were made for each gene for each treatment (no-masking, masking by ZORRO, GBLOCKS, ALISCORE or GUIDANCE), resulting in reconstruction of a total of 10,000 trees (10 genes×2 tree methods×5 treatments×100 replicated simulations). The accuracy of the trees was measured by calculating its unnormalized Robinson-Foulds topological distance to the true tree using the program VANILLA [52].

## Supporting Information

Figure S1 **The 100-taxon tree used to guide the protein sequence simulations.** This tree is derived from the ‘genome tree’ of 720 bacterial species [41] (TreeBase, S10956) as described in Materials and Methods.
(TIF)

File S1 **This file contains all the control files used by ROSE to simulate protein sequence evolution for the ten marker genes.**
(DOC)
