# Supplementary figures and images for: Accounting For Alignment Uncertainty in Phylogenomics
Source: PLoS One. 2012 Jan 17;7(1):e30288. doi: 10.1371/journal.pone.0030288 (PMC3260272; doi:10.1371/journal.pone.0030288)

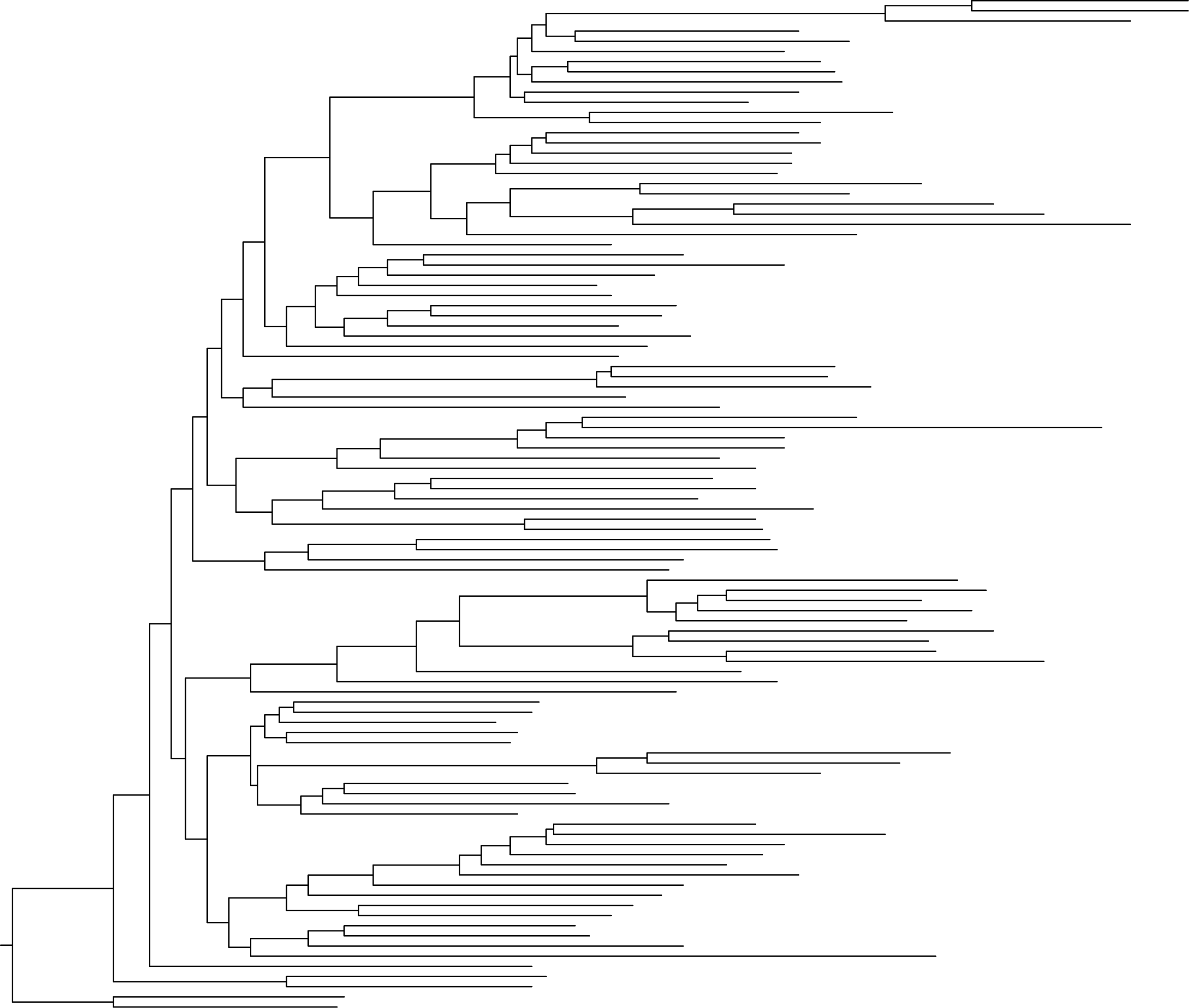

Supplement: Figure S1 — The 100-taxon tree used to guide the protein sequence simulations. This tree is derived from the ‘genome tree’ of 720 bacterial species [41] (TreeBase, S10956) as described in Materials and Methods. (TIF) [file pone.0030288.s001.tif]
